# MRVI1 and NTRK3 Are Potential Tumor Suppressor Genes Commonly Inactivated by DNA Methylation in Cervical Cancer

**DOI:** 10.3389/fonc.2021.802068

**Published:** 2022-01-24

**Authors:** Huihui Ji, Kehan Li, Wenxiao Jiang, Jingwei Li, Jian-an Zhang, Xueqiong Zhu

**Affiliations:** ^1^ Center of Uterine Cancer Diagnosis & Therapy Research of Zhejiang Province, Department of Obstetrics and Gynecology, the Second Affiliated Hospital of Wenzhou Medical University, Wenzhou, China; ^2^ Department of Obstetrics and Gynecology, Taizhou Woman and Children’s Hospital of Wenzhou Medical University, Taizhou, China

**Keywords:** *MRVI1*, *NTRK3*, cervical cancer, TSGs, DNA methylation

## Abstract

The abnormally methylated tumor suppressor genes (TSGs) associated with cervical cancer are unclear. DNA methylation data, RNA-seq expression profiles, and overall survival data were downloaded from TCGA CESC database. DMGs and DEGs were obtained through CHAMP and DESeq packages, respectively. TSGs were downloaded from TSGene 2.0. Candidate hypermethylated/down-regulated TSGs were further evaluated and pyrosequencing was used to confirm their difference in methylation levels of selected TSGs in cervical cancer patients. A total of 25946 differentially methylated CpGs corresponding to 2686 hypermethylated genes and 4898 hypomethylated genes between cervical cancer and adjacent normal cervical tissues were found in this study. Besides, 693 DEGs (109 up-regulated and 584 down-regulated) were discovered in cervical cancer tissues. Then, 192 hypermethylated/down-regulated genes were obtained in cervical cancer compared to adjacent tissues. Interestingly, 26 TSGs were found in hypermethylated/down-regulated genes. Among these genes, low expression of *MRVI1* and *NTRK3* was associated with poor overall survival in cervical cancer. Moreover, GEO data showed that *MRVI1* and *NTRK3* were significantly decreased in cervical cancer tissues. The expression levels of *MRVI1* and *NTRK3* were negatively correlated with the methylation levels of their promoter CpG sites. Additionally, elevated methylation levels of *MRVI1* and *NTRK3* promoter were further verified in cervical cancer tissues by pyrosequencing experiments. Finally, the ROC results showed that the promoter methylation levels of *MRVI1* and *NTRK3* had the ability to discriminate cervical cancer from healthy samples. The study contributes to our understanding of the roles of *MRVI1* and *NTRK3* in cervical cancer.

## Introduction

Cervical cancer is the fourth most common cancer and the fourth leading cause of cancer death in women (1). The prognosis varies depending on the stage of cervical cancer. Compared with patients with early stage of cervical cancer, the five-year survival period of patients with advanced cervical cancer is much shorter (2). Therefore, the identification predictive biomarkers can help effective targeted therapy and treatment decisions.

Epigenetic processes can be reversed and this principle makes it a potential target for therapeutic intervention (3). Epigenetic variations could change the expression of tumor suppressor genes (TSGs) in cervical cancer (4). DNA methylation is an important part of epigenetics (5, 6) and the regulatory effect of DNA methylation on gene expression has been studied extensively (7, 8). DNA methylation levels could be detected by techniques, including pyrosequencing, methylation-specific polymerase chain reaction, methylation-sensitive high-resolution melting, multiplex ligation-dependent probe amplification (MLPA), and Combined bisulfite restriction analysis (COBRA) and MethyLight (9). Aberrant methylation of TSGs could silence the expression of TSGs to consequently promote tumor formation (10). During recent decades, there have been a massive number of studies about TSGs in cervical cancer (11–13). For example, compared with the control samples, the promoter methylation frequency of TSG (including *RARB*, *CADM1*, *PAX1*, and *DAPK1*) in patients with invasive cervical cancer is higher (14). The silencing of TSGs is thought to be an early, driving event in the oncogenic process. Even after human papilloma virus (HPV) clearance, the silencing of TSGs by DNA hypermethylation could trigger carcinogenesis of the cervix (15). However, changes in DNA methylation and related abnormal TSGs expression have not been systematically elucidated in cervical cancer.

Gene methylation profiling and gene expression profiling have been utilized to investigate DNA methylation and gene expression in the molecular mechanism, biological process, and biomarker (16–18). Combined analysis of gene expression and DNA methylation data may contribute to identifying potential biomarkers of cervical cancer for treatment. Therefore, in this study, Illumina HumanMethylation450K methylation data and RNA-seq expression profiles from the Cancer Genome Atlas-Cervical Cancer (TCGA-CESC) were integrated for identifying the DMGs and DEGs in cervical cancer. First, TSGs among hypermethylated/down-regulated genes were found. Second, cervical cancer prognosis-related genes were selected and used as candidate cervical cancer-related TSGs. Then, expression levels of these TSGs were subsequently verified in three independent data sets from the Gene Expression Omnibus (GEO) database. Moreover, cervical cancer tissues and paired adjacent normal cervical tissues were collected to verify the methylation levels of these TSGs. Finally, receiver operating characteristic (ROC) curve analysis was used to assess the development of candidate cervical cancer related TSGs. This study aims to find prognostic and diagnostic TSGs related to cervical cancer through data analysis and experimental verification.

## Results

### Differential Methylation and Expression Analysis

**Figure 1:**
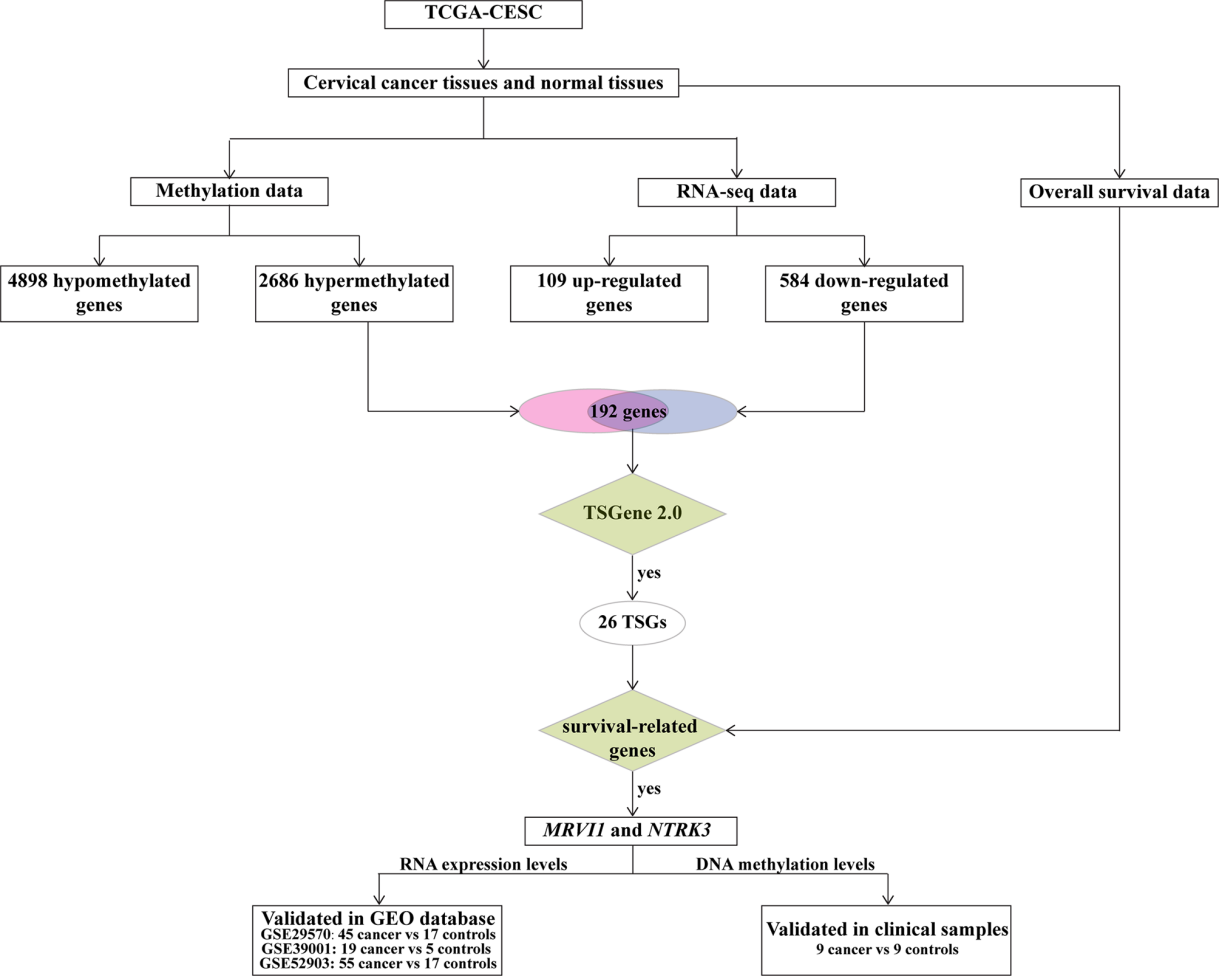
Flow chart of data collection and analysis. TSGs, tumor suppressor genes.

**Figure 2:**
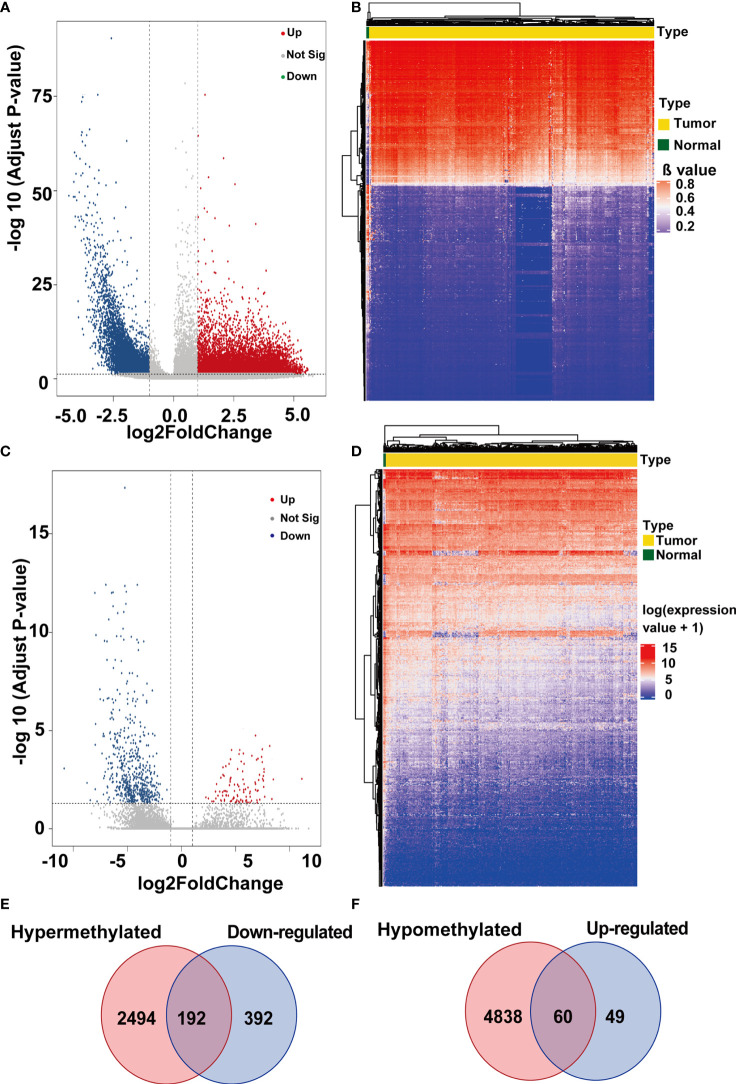
The differentially methylated genes (DMGs) and differentially expression genes (DEGs) from TCGA-CESC database. **(A)** The volcano plot was constructed using log2fold change and −log10 (padj) values. Red plots represent the up-regulated CpG sites, green plots represent the down-regulated CpG sites, and black plots show those CpG sites that are not differentially expressed. **(B)** Heatmap of methylation values for top 1000 CpG sites methylated in cervical cancer patients. CpG sites are shown in the vertical columns and the cervical cancer samples in the horizontal rows. High methylation levels are displayed in red and low methylation levels in blue, according to the scale bar in the right of figure. **(C)** The volcano plot was constructed using log2 fold change and −log10 (padj) values. Red plots represent the up-regulated genes, green plots represent the down-regulated genes, and black plots show those genes that are not differentially expressed. **(D)** Heatmap of methylation values for DEGs in cervical cancer patients. The genes are displayed in the vertical columns and the cervical cancer samples in the horizontal rows. High expression levels are shown in red and low expression levels in blue, according to the scale bar in the right of the figure. **(E)** Venn diagrams of the genes relevant to hypermethylated/down-regulated genes. **(F)** Venn diagrams of the genes relevant to hypormethylated/up-regulated genes.

The workflow of our study is displayed in **Figure 1**. TCGA-CESC was used to identify aberrantly methylation-regulated genes. 12611 hypermethylated CpG sites, which were correspond to 2686 genes, were found in cervical cancer than that in adjacent normal cervical tissues. On the contrary, 13335 hypomethylated CpG sites, which were correspond to 4898 genes, were discovered in cervical cancer compared to adjacent normal cervical tissues (**Figures 2A, B**). Additionally, a total of 693 DEGs (109 up-regulated and 584 down-regulated) were obtained from TCGA-CESC (**Figures 2C, D**). Then, 192 hypermethylated/down-regulated genes (**Figure 2E**) and 60 hypomethylated/up-regulated genes (**Figure 2F**) were identified. Hypermethylated/down-regulated genes were particularly focused in the current study.

### GO and KEGG Pathway Enrichment of the Down-Regulated DEGs With Hypermethylation

**Figure 3:**
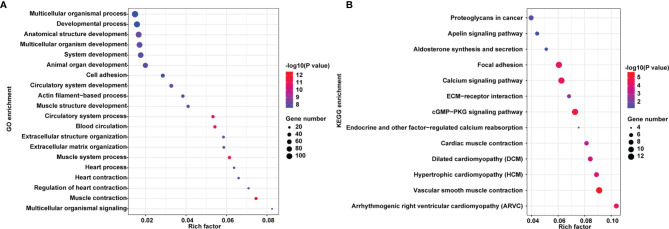
GO and KEGG pathway of the genes relevant to hypermethylation/low expression genes. **(A)** GO of hypermethylated/down-regulated genes. Names of the top 15 items are indicated by the y-axis. The size of the colored dots is the enriched number of genes in each GO classification. The red dots indicate high enrichment and the blue dots indicate low enrichment. FDR value is expressed by the color order on the right edge. **(B)** KEGG pathway enrichment of hypermethylated/down-regulated genes. The size of the dot in the KEGG pathway bubble plot shows the enriched genes. High enriched represented by red, otherwise, by blue.

The top 15 significant GO enrichments of biological processes were illustrated in **Figure 3A**, including extracellular structure organization, multicellular organismal signaling, extracellular matrix organization, and actin filament-based process. There were 13 enrichment pathways, such as vascular smooth muscle contraction, cGMP-PKG signaling pathway, calcium signaling pathway, focal adhesion, ECM-receptor interaction, proteoglycans in cancer, apelin signaling pathway (**Figure 3B**).

### Identification of Candidate TSGs

**Figure 4:**
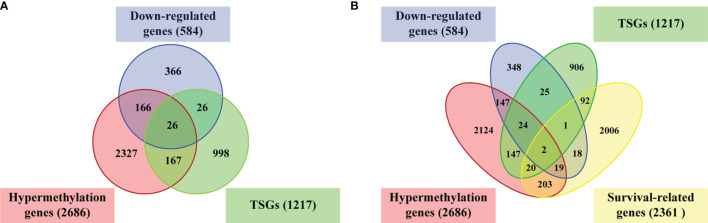
Venn diagram of overlapping genes. **(A)** Venn diagram of overlapping genes in down-regulated genes (blue circle), and hypermethylation genes (red circle), and TSGs (green circle). **(B)** Venn diagram of overlapping genes in down-regulated genes (blue circle), and hypermethylation genes (red circle), TSGs (green circle), and survival-related genes (yellow circle). TSGs, tumor suppressor genes.

26 TSGs were discovered in hypermethylated/down-regulated genes (**Figure 4A**). A total of 2361 cervical cancer survival-related genes were found by Kaplan–Meier analysis using RNA expression data. After integrated TSGs and survival-related genes, 2 overlapping genes (*MRVI1* and *NTRK3*) were discovered and considered as the cervical cancer candidate TSGs (**Figure 4B**).

### Survival Analysis and Validation of *MRVI1* and *NTRK3* Expression

**Figure 5:**
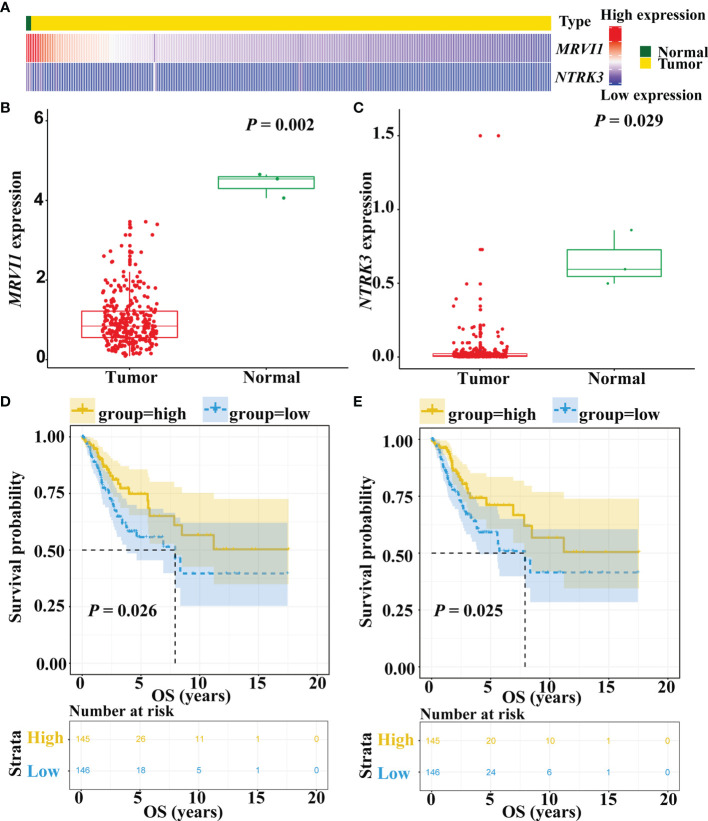
Prognostic values of *MRVI1* and *NTRK3*. **(A)** Hierarchical clustering of *MRVI1* and *NTRK3*; Expression of *MRVI1* **(B)** and *NTRK3* **(C)** in 304 cervical cancer patients and 3 normal cervical tissues. The *MRVI1* and *NTRK3* expression levels (log2(FPKM+1)) are significantly decreased in cervical cancer patients compared with the normal controls. **(D, E)** Kaplan–Meier curves show that low expression of *MRVI1* and *NTRK3* have worse prognosis than that of the high expression of *MRVI1* and *NTRK3*.

The expression levels of *MRVI1* and *NTRK3* genes were obtained in this study (**Figure 5A**). As shown in **Figures 5B, C**, the expression levels of *MRVI1* (*P* = 0.002) and *NTRK3* (*P* = 0.029) were significantly lower in cervical cancer than those in adjacent normal cervical tissues. In addition, patients with low expression of *MRVI1* (*P* = 0.026) and *NTRK3* (*P* = 0.025) had significantly worse survival rates (**Figures 5D, E**).

### Validation of *MRVI1* and *NTRK3* Expression Levels by the GEO Database

**Figure 6:**
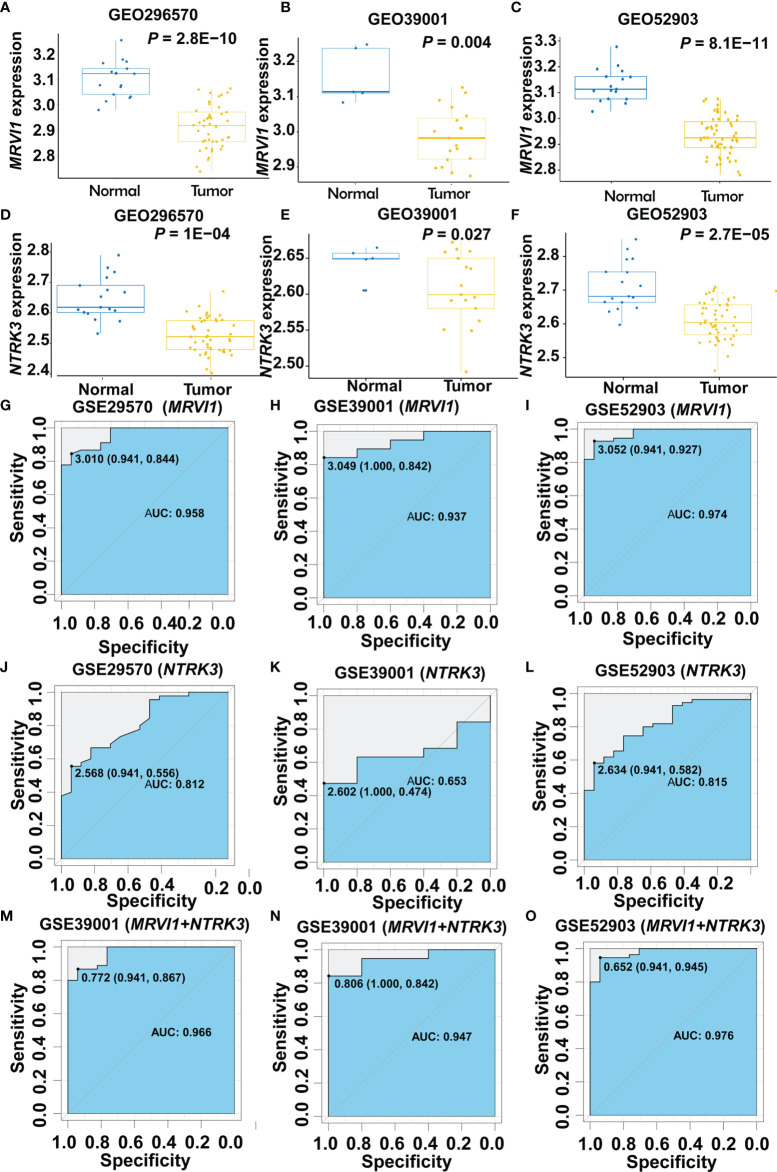
Confirmed expression levels and ROC curve analyses for the prediction of cervical cancer. **(A–F)** Boxplots of gene expression levels in three GEO databases for *MRVI1* and *NTKR3*. The left y-axis shows the mRNA expression levels for *MRVI1* or *NTRK3*. The x-axis represents the two groups (normal tissue and cervical tumor). Each panel represents a different GEO database (GSE29570, GSE39001, and GSE52903). ROC curve of *MRVI1* **(G–I)**, *NTRK1* **(J–L)**, and combined expression **(M–O)** for distinguishing between cervical cancer and non-tumor tissues in individual GEO datasets. AUC, the area under the ROC curve; GEO, Gene Expression Omnibus; ROC, receiver operating characteristics.

*MRVI1* and *NTRK3* levels were all significantly lower in cervical cancers compared to normal cervical tissues in three cervical cancer related datasets (*p* < 0.05, **Figures 6A**–**F**). The ROC curves of *MRVI1* and *NTRK3* gene expression levels to determine cervical cancer were presented in **Figures 6G**–**O**. The ROC curve indicated that *MRVI1* exhibited high diagnostic efficiency for cervical cancer in normal cervical controls in three datasets (AUC > 0.937). The AUC of the prediction model for *NTRK3* was greater than 0.653 in these datasets. The AUC of the combined prediction model of *MRVI1* and *NTRK3* (AUC > 0.947) was higher than that of the *MRVI1* (AUC > 0.937) and *NTRK3* (AUC > 0.653) (**Figures 6G**–**O** and **Table 1**). These results suggested that the expression levels of *MRVI1* and *NTRK3* could distinguish between cervical cancer patients and healthy controls.

**Table 1:** Accuracy of *MRVI1* and *NTRK3* for predicting the prognosis of cervical cancer patients.

	GEO	AUC	95% CI	Cut off point	Sensitivity	Specificity
*MRVI1*	GSE29570	0.958	0.914-1.000	3.010	0.844	0.941
	GSE39001	0.937	0.842-1.000	3.049	0.842	1.000
	GSE52903	0.974	0.944-1.000	3.052	0.927	0.941
*NTRK3*	GSE29570	0.812	0.698-0.925	2.568	0.556	0.941
	GSE39001	0.653	0.432-0.872	2.602	0.474	1.000
	GSE52903	0.815	0.711-0.919	2.634	0.582	0.941
Combination	GSE29570	0.966	0.928-1.000	0.772	0.867	0.941
	GSE39001	0.947	0.861-1.000	0.806	0.842	1.000
	GSE52903	0.976	0.947-1.000	0.652	0.945	0.941

AUC, area under curve; 95% CI, 95% confidence interval.

### Correlation Analysis of Promoter Region Methylation Level and Gene Expression Level

**Figure 7:**
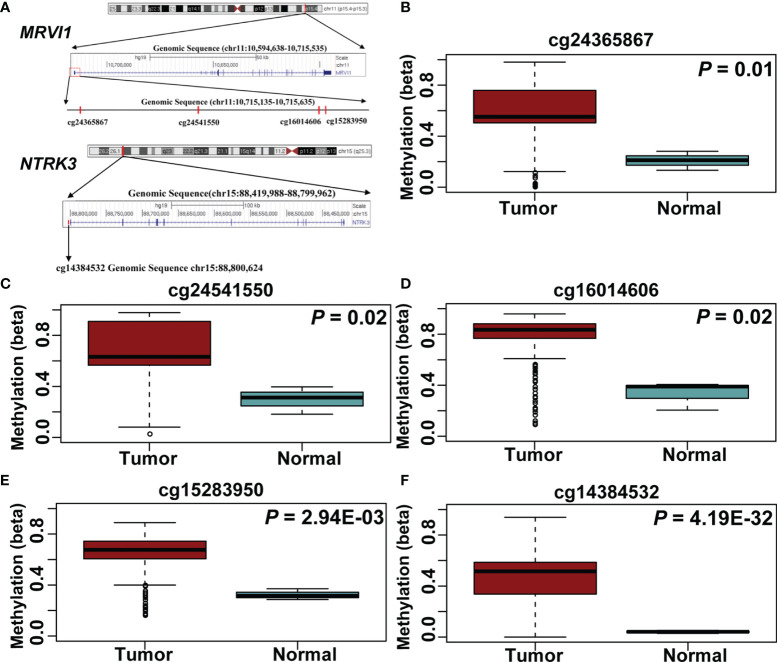
Five CpG sites in *MRVI1* and *NTRK3* promoter regions. **(A)** The locations of CpG sites. The level of *MRVI1* promoter DNA methylation **(B–E)** and *NTRK3* promoter DNA methylation **(F)** presented as a box plot in the cervical cancer and normal cervical tissues.

**Figure 8:**
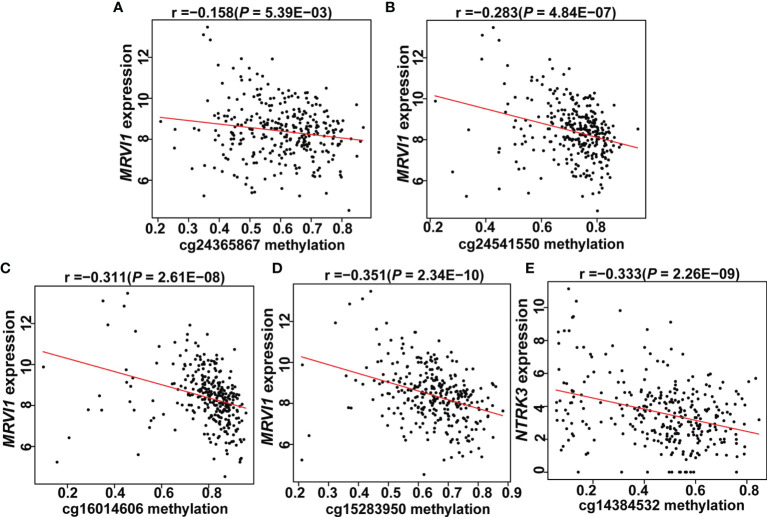
Expression of two genes correlated with promoter CpG site methylation in cervical tissues. **(A–D)** *MRVI1* gene expression was negatively correlated with DNA methylation levels of cg24365867, cg24541550, cg16014606, and cg15283950. **(E)** *NTRK3* gene expression was negatively correlated with DNA methylation level of cg14384532. x-axis: DNA methylation levels; y-axis: relative gene expression levels; red line: linear regression.

A total of 5 CpG sites were located in the promoter regions of *MRVI1* (cg24365867, cg24541550, cg16014606, and cg15283950) and *NTRK3* (cg14384532) (**Figure 7A**). The methylation levels of these CpG sites were up-regulated in cervical tumors compared to controls (*p* < 0.05, **Figures 7B**–**F**). Further correlation analysis showed that the methylation levels of these CpG sites were negatively associated with gene expression for these two genes (*p* < 0.05, **Figure 8**).

### Verification of Differences in Promoter Methylation Levels of *MRVI1* and *NTRK3* by Pyrosequencing Experiments

**Figure 9:**
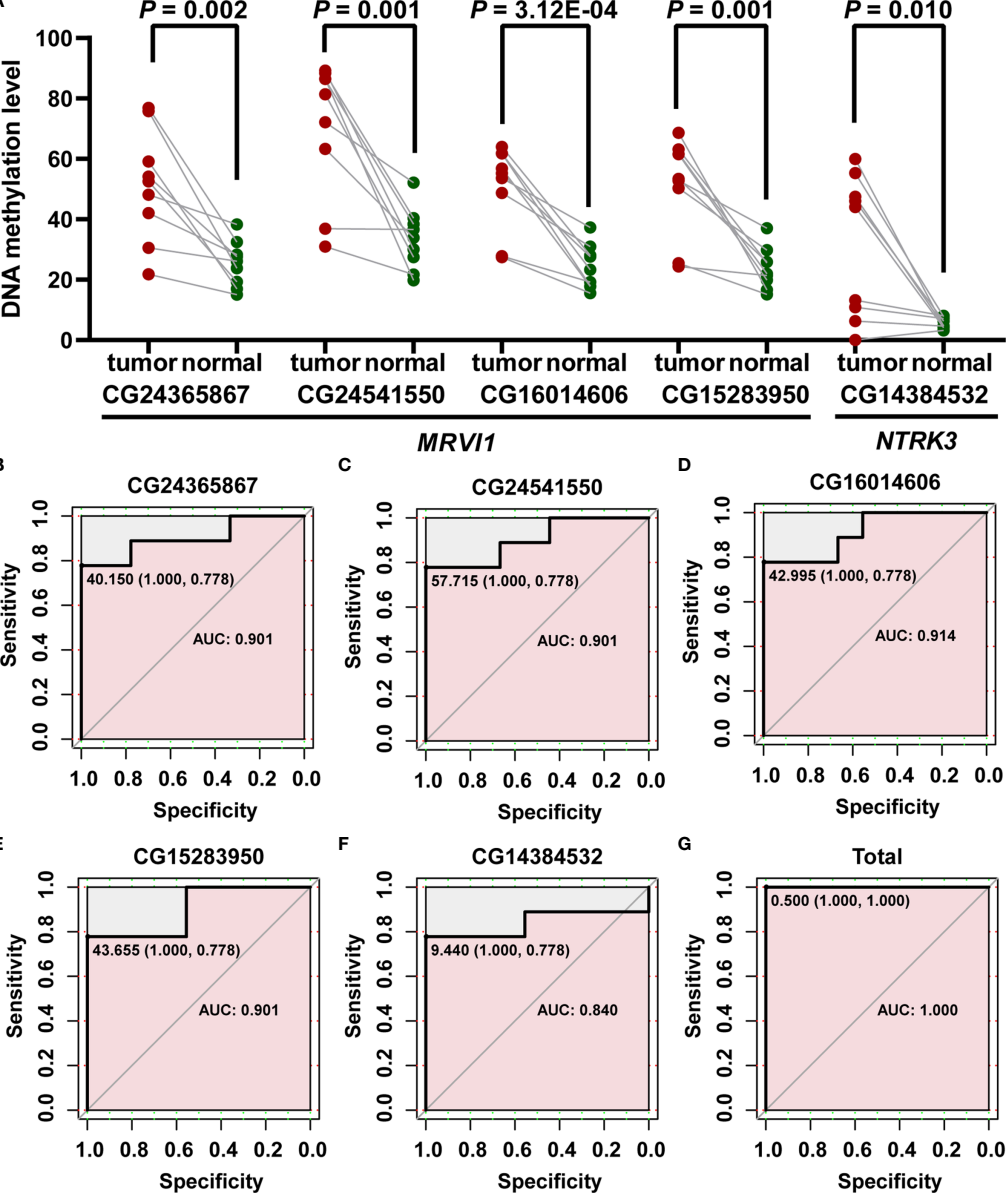
Confirmed methylation levels and ROC curve analyses for the prediction of cervical cancer. **(A)** The methylation levels of *MRVI1* and *NTRK3* were significantly higher in cervical cancer tissues than in adjacent normal tissues. ROC curve of methylation levels of *MRVI1* **(B–E)**, *NTRK1* **(F)**, and combined methylation levels of five CpGs **(G)** for distinguishing between cervical cancer and non-tumor cervical tissues. AUC, the area under the ROC curve; ROC, receiver operating characteristics.

In order to verify the differential methylation levels of *MRVI1* and *NTRK3* between cervical cancer and adjacent normal cervical tissues, pyrosequencing experiments were conducted. As shown in **Table 2** and **Figure 9A**, the methylation levels of cg24365867, cg24541550, cg16014606, and cg15283950 of *MRVI1* gene in cervical cancer were significantly higher than that in adjacent normal cervical tissues (*p* < 0.05). Compared with adjacent normal tissues, a significantly elevated methylation level of cg14384532 on *NTRK3* was also found in cervical cancer tissues (*p* < 0.05, **Table 2** and **Figure 9A**). In addition, ROC analysis was performed and AUC was calculated to assess the potential diagnostic value of *MRVI1* and *NTRK3* using the methylation levels of CpG sites on promoter regions. As shown in **Figures 9B**–**G**, five CpG sites had excellent diagnostic performance for discriminating cervical cancer from healthy cervical samples (cg24365867, *p* = 0.003, AUC = 0.901; cg24541550, *p*= 0.003, AUC = 0.901; cg16014606, *p* = 0.002, AUC = 0.914; cg15283950, *p* = 0.003, AUC = 0.901; cg14384532, *p* = 0.014, AUC = 0.840; Combined, *p* = 0.0002, AUC = 1.000).

**Table 2:** Association between *MRVI1* and *NTRK3* methylation status and cervical cancer.

		Tumor	Non-tumor	*P* value
*MRVI1*	CG24365867	51.21 ± 18.44	25.30 ± 7.47	0.002
	CG24541550	70.54 ± 22.47	33.37 ± 10.04	0.001
	CG16014606	50.81 ± 13.95	24.19 ± 7.27	0.0003
	CG15283950	51.26 ± 16.02	23.69 ± 6.81	0.001
*NTRK3*	CG14384532	31.47 ± 23.43	5.62 ± 1.73	0.010

## Discussion

Cervical cancer is one of the most common types of cancer and represents a major global health challenge (1). Since aberrant DNA methylation occurs very early during tumorigenesis (19), it could therefore be used as an early diagnostic biomarker (20). In this study, hypermethylated and significantly lower expressions of TSGs *MRVI1* and *NTRK3* were discovered in cervical cancers than that in normal cervical tissues using the bioinformatics. The differences of *MRVI1* and *NTRK3* expressions between cervical cancer specimens and normal cervical tissues were further verified *via* three GEO datasets. Besides, the low expression of *MRVI1* and *NTRK3* was negatively associated with high methylation levels of promoter CpG sites. Moreover, promoter hypermethylation levels of *MRVI1* and *NTRK3* were also found in our clinical cervical cancer samples. ROC curve analyses proved the diagnostic value of *MRVI1* and *NTRK3* in cervical cancer. Furthermore, low expression of *MRVI1* and *NTRK3* was associated with poor prognosis of cervical cancer. These results enhanced our understanding of the DNA methylation pattern of TSGs in cervical cancer.

*MRVI1* is a protein-coding gene, which has been widely studied in cancer (21, 22). *MRVI1* was reported to regulate the cellular release of calcium signal (23), which plays an important role in cancer cell proliferation invasiveness (24). One study discovered that MRVI1 was transcriptionally activated by p53, and p53-induced inhibition of colorectal cancer prognosis was depended on MRVI1 (25). Zhu et al. found that the *MRVI1*-AS1/ATF3 signalling pathway could increase paclitaxel chemosensitivity by modulating the Hippo-TAZ signalling pathway in nasopharyngeal cancer (21). Another research found that miR-940 could promote proliferation and metastasis of endometrial carcinoma through the regulation of *MRVI1* (22). High expression of *MRVI1* had a better prognosis than that of the low expression of *MRVI1* in endometrial carcinoma (22). Unfortunately, the role of *MRVI1* in cervical cancer has not yet been reported. In the current study, the overall survival of cervical cancer patients with low *MRVI1* expression was also significantly shorter than those with high *MRVI1* expression, which is consistent with previous endometrial carcinoma study.

*NTRK3* encodes the TrkC protein, a member of neurotrophic tropomyosin receptor kinase (Trk) family, which autophosphorylates and motivates various signalling pathways such as MAPK and PI3K/AKT pathways (26). Trk aberrations, including gene fusion, gene overexpression, and single nucleotide variation, are involved in the pathogenesis of many cancers, among which NTRK3 gene fusion is extremely confirmed for oncogenic event (27). Unusual activation of NTRK3 and its fusion proteins may balance the epithelial–mesenchymal transition (EMT), oncogenicity, and tumor growth rate *via* triggering various signalling pathways (28). *ETV6*-*NTRK3* gene fusion acted as a potent oncogene driver and had been presented in the majority of cases of infantile fibrosarcoma (29). Oncogenic fusions in *NTRK* family receptor tyrosine kinases had been identified in several cancers and could serve as therapeutic targets, for instance in spitz tumors (30), fibrosarcoma (31), gastrointestinal stromal tumors (32), and inflammatory myofibroblastic tumors (33). Conversely, *NTRK3* expression was a good prognosis factor in a variety of cancers and more specifically in melanomas (34), neuroblastomas (35), and colorectal cancer (36). *NTRK3* expression and activation had been shown to trigger apoptosis in medulloblastoma cells (37). In recent years, *SPECC1L*-*NTRK3* gene fusion was found in cervical sarcoma patients (38). However, the research on *NTRK3* gene in cervical cancer is rare. Depending on the present study, *NTRK3* expression was significantly lower in cervical cancer specimens than that in normal cervical tissues, and low *NTRK3* expression was associated with a poor prognosis. These findings suggested that *NTRK3* might likewise serve as a tumor suppressor gene in cervical cancer.

In present study, two TSGs (*MRVI1* and *NTRK3*) were identified *via* bioinformatics. Nevertheless, a total of 26 hypermethylated/down-regulated TSGs have been discovered, the rest TSGs should be further studied. Although, the hypermethylation levels of *MRVI1* and *NTRK3* were verified in 9 cervical cancer tissues by pyrosequencing, the large number of clinical samples should be collected in further study. Hypermethylated and down-regulated expression levels of TSGs *MRVI1* and *NTRK3* have been identified in the current study; however, the detail epigenetic regulatory mechanism under cervical cancer still needs further investigation. In summary, our results revealed that hypermethylation in the promoter regions of *MRVI1* and *NTRK3* genes might lead to low expression in cervical cancer. Low expression levels of *MRVI1* and *NTRK3* were associated with poor prognosis of cervical cancer. The methylation levels and expression levels of *MRVI1* and *NTRK3* had the ability to effectively discriminate cervical cancer from healthy samples. Therefore, *MRVI1* and *NTRK3* genes may play important roles in the occurrence and prognosis of cervical cancer. It could be further explored and validated as a therapeutic target for cervical cancer. In conclusion, the down-regulation of *MRVI1* and *NTRK3* may drive cervical cancer through hypermethylation of their promoters. Further studies are needed to draw more attention to the roles of these TSGs in cervical cancer.

## Materials and Methods

### Data Resources for DNA Methylation, RNA-Seq Data and Clinical Information

Illumina Infinium HumanMethylation450K and RNA-seq expression profiles were downloaded from TCGA-CESC (https://cancergenome.nih.gov/). Methylation data of 307 cervical cancer and 3 normal cervical tissues were collected in the present study. The probes were annotated by using the Bioconductor package with the human genome assembly GRCh37 (hg19). Gene expression profile corresponding to abnormally methylated genes was also download. In addition, the corresponding clinical overall survival data of 291 samples were included. Besides, 3 cervical cancer-related expression datasets (GSE29570, GSE39001, and GSE52903) were obtained from the GEO database. GSE29570 (39) includes the expression data from 17 healthy female exocervix samples and 45 cervical cancer samples, GSE39001 (40) contains data from 5 healthy female cervical samples and 19 cervical cancer samples, and GSE52903 (41) contains expression profiles from a discovery cohort of 17 healthy female cervical samples and 55 cervical cancer samples.

### Differential Methylation and Gene Expression Analysis

Between cervical cancer tissues and normal cervical tissues, significant DMGs and DEGs were identified using DESeq package (42) and CHAMP package of R (43), respectively. The false discovery rate (FDR) was adopted to avoid the occurrence of false-positive results. FDR < 0.05 and |Log2 Fold change (Log2FC)| > 1 were used to select significant DMG or DEG.

### Gene Ontology (GO) and KEGG Pathway Analysis

GO and KEGG pathway enrichment analysis of hypermethylated/down-regulated genes was performed using the g:Profiler program (44).

### Searching for TSGs Associated With Cervical Cancer

Among hypermethylated/down-regulated genes, TSGs were identified based on TSGene 2.0 (45). With the median expression level as the demarcation point, 291 patients with clinical data in TCGA were divided into low-risk group and high-risk group. Kaplan-Meier analysis in the survival package of R (46) was used to compare the difference in overall survival between the two groups. Prognostic-related TSGs were considered as candidate genes for cervical cancer. To solve the problem of a small number of normal tissues in TCGA-CESC, the expression levels of cervical cancer candidate TSGs in three GEO datasets (GSE29570, GSE39001, and GSE52903) were further compared by T test using R.

### Pyrosequencing Experiment

Nine pairs of cervical cancer specimens and adjacent normal cervical tissues were obtained from the Second Affiliated Hospital of Wenzhou Medical University. Cervical cancer patients were diagnosed by experienced pathologists based on the results of surgically removed specimens (**Supplementary Table 1**). Human genomic DNA was extracted from tissue samples using the Genomic DNA Extraction Kit (Qiagen, Dusseldorf, Germany). DNA concentrations were determined by the Infinite F200 Tecan microplate reader (Tecan, männedorf, Switzerland). Primers were designed using the PyroMark Assay Design Software 2.0 and bisulfite-treated DNA PCR-amplified using the PyroMark PCR kit prior to analysis on a PyroMark Q96 according to manufacturer’s instruction (Qiagen, Dusseldorf, Germany). Sequences of the PCR primers were shown in **Table 3**. Amplification was carried out as follows: 95°C for 3 min, followed by 40 cycles of 94°C for 30 s, 56°C for 30 s, and 72°C for 1 min, with a final elongation step at 72°C for 7 min. Raw data were analyzed using PyroMark Q96 software (Qiagen, Dusseldorf, Germany). The research protocol was approved by the Ethics Committee of the Second Affiliated Hospital of Wenzhou Medical University. Written informed consents were obtained from all subjects.

**Table 3:** Primers used for the pyrosequencing assay.

Gene	Forward primer (5’ to 3’)	Reverse primer (5’ to 3’)	Sequencing primer (5’ to 3’)
*MRVI1*	GGGGATTGTTATTTGTTGTGTGGATAT	Biotin- AAACTCCTCTAAAAACCCCACTC	AGGGGTTTAGGGTGA
	TGGGGAGTTTTTATTATTTAAGGTTAATG	Biotin- TAAATCCCAACCCCTCTCAAA	AGGTTAATGTTATATTTGGTTT
	AAGTATGTGAGTTTGGAGAAGA	Biotin- TCTCCCAAACCATTCTCTCTAAC	GGTAGGGGTTGTTTTTA
*NTRK3*	GGAAATGAGTGTTATTTAGATTAAGGAT	Biotin- ACCCCAAAAAAACACCCA	AGTGTTATTTAGATTAAGGATT

### Statistical Analysis

Pearson correlation coefficient was used to correlate promoter methylation levels with candidate TSGs expression levels. ROC curves were used to compare the sensitivity and specificity of the candidate TSGs expression levels and promoter methylation levels in the prediction of cervical cancer. All the data were analyzed using R scripts. A two-tailed *p* value < 0.05 was considered statistically significant.

## Data Availability Statement

The raw data supporting the conclusions of this article will be made available by the authors, without undue reservation.

## Author Contributions

Conceptualization, HJ and XZ. Methodology, KL. Formal analysis, KL, WJ, JL, J-aZ. Investigation and writing, HJ and XZ. Visualization and supervision, XZ. All authors have read and agreed to the published version of the manuscript.

## Funding

This work was supported by the grant from the Subject of Integrated Traditional Chinese and Western Medicine in Zhejiang Province (2017-XK-A42). The study sponsors had no involvement in the collection, analysis, and interpretation of data, or in the writing of the manuscript.

## Conflict of Interest

The authors declare that the research was conducted in the absence of any commercial or financial relationships that could be construed as a potential conflict of interest.

## Publisher’s Note

All claims expressed in this article are solely those of the authors and do not necessarily represent those of their affiliated organizations, or those of the publisher, the editors and the reviewers. Any product that may be evaluated in this article, or claim that may be made by its manufacturer, is not guaranteed or endorsed by the publisher.
