# Supplementary material for: MRVI1 and NTRK3 Are Potential Tumor Suppressor Genes Commonly Inactivated by DNA Methylation in Cervical Cancer
Source: Front Oncol. 2022 Jan 24;11:802068. doi: 10.3389/fonc.2021.802068 (PMC8818726; doi:10.3389/fonc.2021.802068)
Supplement: Supplementary file 1 [file Table_1.doc]

**Supplemental Table 1: The clinico-pathological features of the cervical cancer patient samples used in this study.**

| Item | n |
| --- | --- |
| **Age，years** |  |
| ≤50 | 4 |
| ＞50 | 5 |
| **Histologic subtype** |  |
| Squamous cell carcinoma | 5 |
| Adenocarcinoma | 1 |
| Adenosaquamous carcinoma | 3 |
| **HPV status** |  |
| Genotype 16 | 3 |
| Genotype 18 | 1 |
| Other Genotype | 1 |
| unknown | 4 |
| **Lymph node** |  |
| Yes | 0 |
| No | 9 |
